# Achieving timely treatment for early-stage non–small cell lung cancer: Factors associated with delayed surgical resection and proposed quality benchmarks

**DOI:** 10.1016/j.xjon.2026.101682

**Published:** 2026-02-16

**Authors:** Haley I. Tupper, Joyita Bhattacharjee, Varada Sarovar, Dominic Amara, Julie A. Schmittdiel, Charles P. Quesenberry, Amy L. Cummings, Jeffrey B. Velotta, Lori C. Sakoda

**Affiliations:** a Division of General Surgery, Department of Surgery, University of California, Los Angeles, Los Angeles, Calif; b Division of Research, Kaiser Permanente Northern California, Pleasanton, Calif; c Division of Hematology-Oncology, Department of Medicine, University of California, Los Angeles, Los Angeles, Calif; d Division of Thoracic Surgery, Department of Surgery, Kaiser Permanente Northern California, Oakland, Calif; e Department of Surgery, University of California San Francisco School of Medicine, San Francisco, Calif; f Department of Clinical Science, Kaiser Permanente Bernard J. Tyson School of Medicine, Pasadena, Calif; g Department of Health Systems Science, Kaiser Permanente Bernard J. Tyson School of Medicine, Pasadena, Calif

**Keywords:** non–small cell lung cancer, health care quality, surgery, timely care, delayed treatment, quality metrics

## Abstract

**Objective:**

Timely care is a key quality indicator. Resection delayed beyond 8 weeks for early-stage non–small cell lung cancer (NSCLC) negatively impacts prognosis, yet diagnosis-to-treatment time has steadily increased. We sought to identify drivers of delayed surgical resection and generate evidence for timeliness metrics for optimal outcomes.

**Methods:**

We evaluated patient-level factors associated with delayed versus timely surgery (>8 vs ≤8 weeks after diagnosis) for clinical stage I-II NSCLC between 2009 and 2019. Factors were identified by estimating adjusted relative risks (aRRs) and 95% CIs using modified Poisson regression. Time to process-level steps from diagnosis, along with the number and combination of these steps, were examined by timeliness of surgery.

**Results:**

Among 2567 patients, 46.0% received timely surgery. Factors associated with delayed surgery included Black (aRR, 1.14; 95% CI, 1.02-1.27) and Asian (aRR, 1.14; 95% CI, 1.00-1.29) race, distance to surgical facility of >50 miles (aRR, 1.23; 95% CI, 1.08-1.40), and greater health care use (≥25 visits, aRR, 1.72; 95% CI, 1.53-1.93). More preoperative steps increased time to surgery, but no individual step drove delays. When performed, preoperative steps occurred within the following intervals in 75% of timely surgery recipients: positron emission tomography-computed tomography: 21 days, biopsy: 20 days, pulmonary function tests: 28 days, and thoracic surgery consult: 30 days.

**Conclusions:**

As health system and oncologic care processes grow in complexity, timely treatment warrants greater attention. Our results from an integrated health system indicate several patient- and process-level factors contribute to delays that can be mitigated and offer preliminary benchmarks to promote timely NSCLC management.

## Graphical Abstract

**Figure:**
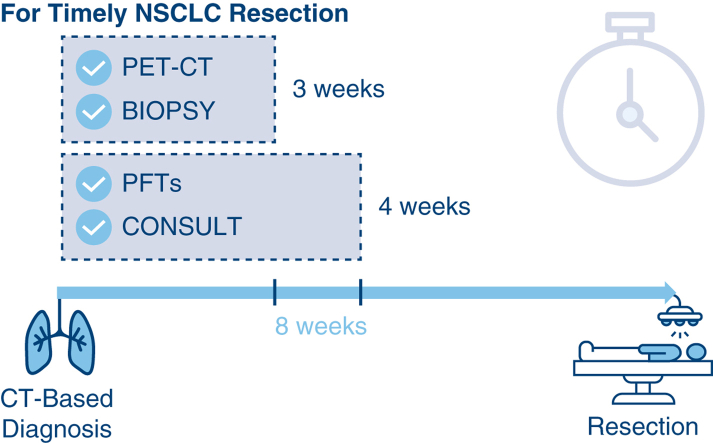

**Figure:**
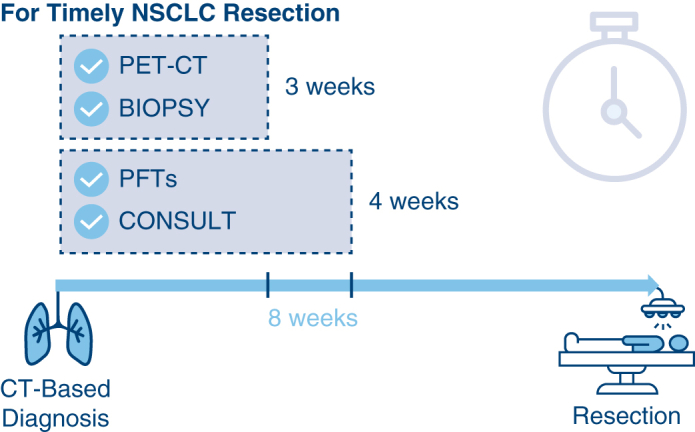
Recommended time intervals from CT-based diagnosis to achieve timely NSCLC care.

**Central Message**

Patient factors and more preoperative workup steps increase treatment delay. Benchmarks to facilitate timely NSCLC resection are PET-CT and biopsy in 3 weeks and PFT and surgery consult in 4 weeks.

**Perspective**

Resection delayed >8 weeks for early-stage NSCLC negatively impacts prognosis, yet diagnosis-to-treatment time is steadily increasing. Social determinants, like race, and treating facility distance may increase delays. More preoperative steps also increase time to surgery. These benchmarks may facilitate timely resection: PET-CT and biopsy in 3 weeks and PFT and surgery consult in 4 weeks.

The National Academy of Medicine defines quality health care by 6 domains: patient-centered, effective, safe, efficient, equitable, and timely.^1^ Greater attention has been paid to effectiveness and safety, whereas equity and timeliness have been underemphasized. Simultaneously, the management of cancer has become increasingly complex and multidisciplinary, even for early-stage disease, with accompanying risks of delayed treatment and loss to follow-up. Median time from lung cancer diagnosis to treatment initiation has progressively increased over the last 3 decades, with a National Cancer Database (NCDB) study reporting the median time had increased 6 days between 2010 and 2018.^2^, ^3^, ^4^

Previously, we demonstrated that curative-intent surgery delayed beyond 8 weeks of clinical radiologic diagnosis of non–small cell lung cancer (NSCLC) is associated with increased risk of 5-year mortality and 1-year lung cancer recurrence. Many factors can delay care, including patient-, provider-, and process-level factors, which can include clinical and administrative processes that are intra- and interorganizational.

To achieve timely care, greater understanding of contributing factors to diagnosis-to-treatment delays, especially ones that are modifiable or inequitable, is critical. Some delays may have clinically appropriate drivers, such as complete staging work-up, whereas others may have inappropriate drivers, such as disparate access to care. Furthermore, as cancer diagnosis, staging, and treatment becomes increasingly complex and multistep, it is worthwhile to consider how apparently clinically appropriate drivers may be delaying care to improve clinical practice. Interventions can also be developed to mitigate inappropriate drivers.

Most databases, including the NCDB, lack the requisite data to adequately study preoperative processes. Even institutional databases are commonly limited by preoperative care fragmentation across organizations. A closed health care delivery system with integrated payor-provider functions is ideal for evaluating patient-level and intraorganizational process-level delays in treatment. This reduces the noise from interorganizational delays, such as prior authorization processes, incomplete or fragmented care pathways, or communication breakdown between organizations.^3^ The objective of this study was to identify patient-level and process-level drivers of delayed surgical resection after diagnosis of early-stage NSCLC and preliminary timeliness benchmarks for composite preoperative workup steps.

## Methods

### Study Population

Linking institutional cancer registry and electronic health record databases, we identified adults diagnosed with clinical stage I-II NSCLC who underwent elective curative-intent surgical resection between 2009 and 2019. Patients who received neoadjuvant chemotherapy, lacked appropriate preoperative imaging (defined as a computed tomography [CT] and/or positron emission tomography [PET]-CT of the chest within 6 months of surgery), were aged 85 and older, or had less than 1 year of continuous institutional health plan membership before surgery were excluded. As previously described,^5^ date of diagnosis was uniformly defined as the first suspicious finding on diagnostic CT of the chest that triggered comprehensive diagnostic and staging workup.

### Setting

This study was conducted in a large, integrated health care system that operates as a health plan and provider, facilitating functional coordination. Care is provided under a single organization with unified health information technology with regional oversight. This system provides health care to approximately 4.5 million members, nearly one third of Northern California's population, through 21 hospitals with affiliated outpatient clinics. Thoracic surgery was geographically centralized to 4 hospitals with a hub-and-spoke model in 2014, but patients can have preoperative consultations either in-person or virtually. Radiology, pulmonology, and oncology care remain decentralized. Multidisciplinary tumor boards were established at each thoracic surgery hub in 2017. The Kaiser Permanente Northern California Institutional Review Board approved conduct of this minimal risk study with a waiver of informed consent (no. 1610759; initial approval date: September 16, 2021).

### Data Sources and Variables

**Figure E1:**
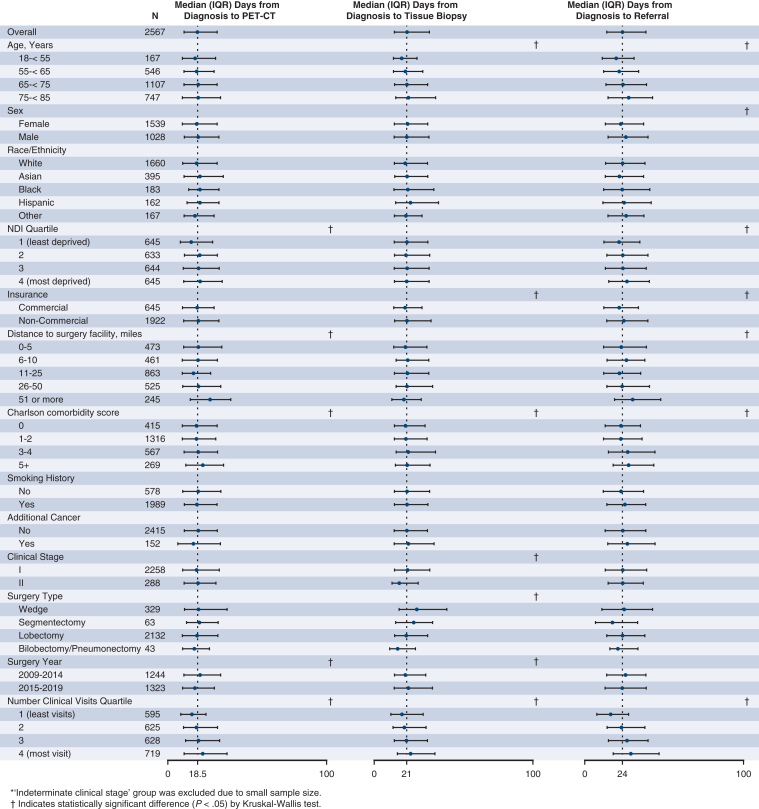

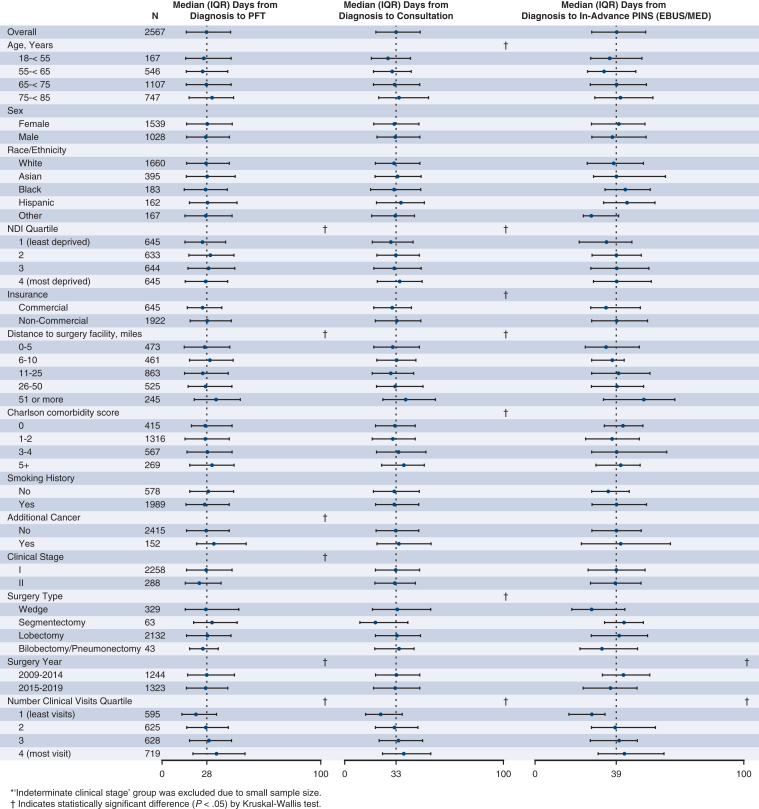

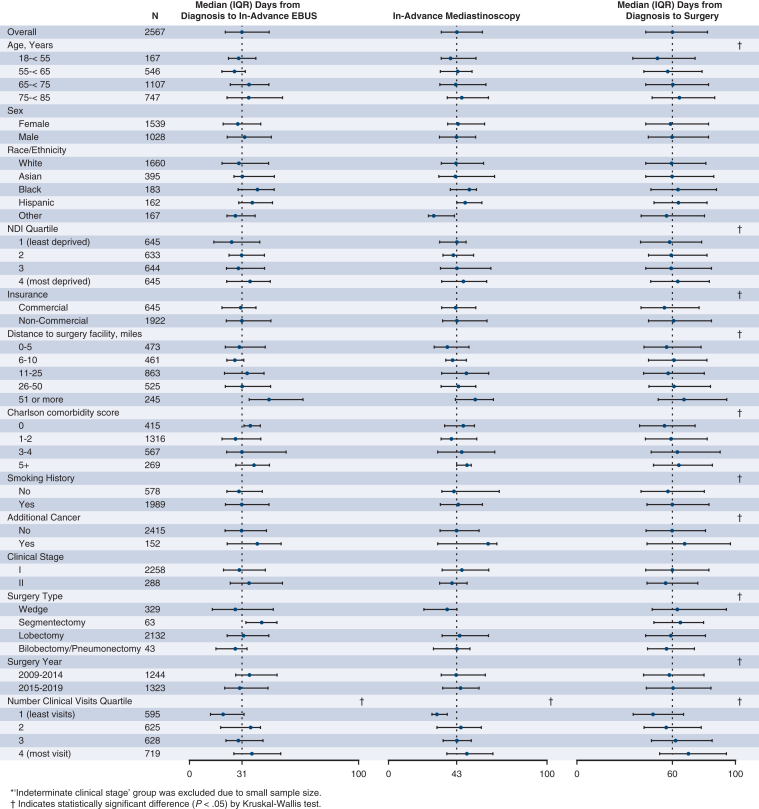
Median time from diagnosis to individual step completion time by patient-level factors (including referral, tissue biopsy, PINS: endobronchial ultrasound and mediastinoscopy). *PINS*, Preoperative invasive nodal staging; *IQR*, interquartile range; *PET*, positron emission tomography; *CT*, computed tomography; *PFT*, pulmonary function text; *NDI*, Neighborhood Deprivation Index.

The following patient-level demographic, socioeconomic, and clinical characteristics were extracted from electronic health record databases as of surgery date: age, sex, race/ethnicity, Neighborhood Deprivation Index (NDI), insurance status, driving distance to surgical facility, Charlson Comorbidity Index (CCI) score, smoking history, other non-NSCLC cancer history, clinical NSCLC stage, surgery type, surgery year, and number of clinical visits in the past 6 months. The first receipt of the following process-level steps were collected between CT-based diagnosis date and surgery date: PET-CT, pulmonary function tests (PFTs), thoracic surgery consult, and biopsy (ie, tissue biopsy and/or preoperative invasive nodal staging). Information on thoracic surgery referral and disaggregated information on biopsy are provided in the Figure E1. Tests that occurred before diagnosis (eg, PFTs) and preoperative invasive nodal staging procedures that occurred during the same anesthetic as surgery are also presented in the Figure E1; presumably, steps that occur before diagnosis or on the same day as surgery do not meaningfully contribute to treatment delay. Data were ascertained from institutional administrative and clinical databases, including the institutional cancer registry, which adheres to US Surveillance, Epidemiology, and End Results program data standards. The completeness and accuracy of the following variables were verified through structured chart review: CT-based diagnosis date, smoking history, staging procedures (PET-CT, PFTs, tissue biopsy, invasive nodal staging), receipt of surgical resection for curative intent, and date and type of surgery.

The methodology for defining radiographic-based diagnosis date is described in depth elsewhere.^5^ In brief, the diagnosis date was uniformly defined as the diagnostic CT date that immediately preceded appropriate diagnostic and staging work-up for NSCLC, indicating radiographic findings had reached a certain clinical suspicion threshold. It is possible that additional CTs, beyond PET-CT, were performed between this radiographic diagnosis date and surgery. Clinically documented race and Hispanic ethnicity were aggregately reported; if the patient identified as Hispanic, race was not reported. NDI was divided into ordinal quartiles, where 1 is least deprived. Insurance was categorized dichotomously as commercial and noncommercial (Medicaid, Medicare, and other). Driving distance was calculated from the patient's primary residence and then categorized on the basis of commonly available options on health care locators. CCI, which can range from 0 to 24, was categorized as 0 (none), 1-2 (mild), 3-4 (moderate), and 5+ (severe). Patients with an additional non-NSCLC diagnosis in their electronic health record before surgery were described as having “additional cancer.” The corresponding AJCC staging system (version 6, 7, or 8) for the year of NSCLC diagnosis was used for the clinical stage and patients with “Nx” or discrepant clinical nodal stages were classified as “indeterminate.” For surgery type, bilobectomy and pneumonectomy were combined due to small numbers. Year of surgery was categorized into 2009-2014 and 2015-2019 to account for the regionalization of thoracic surgical care in 2014, as described previously. The number of clinical visits, as a measure of health care use, was categorized into ordinal quartiles. Clinical encounters included ambulatory or urgent care visits, virtual visits, emergency department visits, laboratory test-only encounters, radiology encounters, and inpatient hospitalizations. Although diagnostic and staging work-up for lung cancer generally follows a replicable pattern, the completion and sequencing of preoperative tests is not uniform. Consequently, time intervals for the completion of each preoperative step (eg, PET-CT, PFT) were systematically defined from diagnosis date. All time intervals were measured in days. Given delays may be additive, the number of preoperative steps received were quantified and the unique step combinations for each “preoperative step volume” (ie, 1, 2, 3, or 4 preoperative steps) were characterized irrespective of sequence. Our primary outcome was delayed surgery, meaning surgery performed more than 8 weeks after diagnosis.^5^

### Statistical Analysis

Statistical analysis was performed using SAS, version 9.4, and R, version 4.3.1. We performed modified Poisson regression to estimate the adjusted relative risk (aRR) with 95% CIs of delayed surgery associated with each patient-level factor of interest. Using the Wilcoxon rank-sum and Pearson ^χ2^ tests as appropriate, we compared measures of central tendency (median and interquartile range [IQR]) for (1) time to completion for each individual step by patient-level factors; (2) time to completion for each individual step, stratified by delayed versus timely surgery; and (3) time to completion for each “preoperative step volume” and step combination. To explore potential variation arising from centralization of thoracic surgery and use of multidisciplinary tumor boards, we repeated analyses limited to patients who underwent surgery during those time periods (2015-2019; 2018-2019).

## Results

Our analyses included 2567 patients, with 54.0% receiving surgery more than 8 weeks after diagnosis. The median age was 70.2 years [IQR, 64.1-75.8]; 60.0% were female, 35.3% were non-White, and 74.9% had noncommercial insurance. The majority (70.0%) lived within 25 miles of their surgery facility, with a median driving distance of 15.3 miles. Most had at least 1 comorbidity (CCI 1+: 83.8%), a history of smoking (77.5%), and clinical stage I disease (88.0%) (Table 1). Patients had a median of 19.0 [IQR, 15.0-24.0] clinical encounters before surgery.

**Table 1:** Risk of delayed surgery for early-stage NSCLC associated with patient-level factors

Variable	Overall (N = 2567)	≤8 weeks (timely) (n = 1182)	>8 weeks (delayed) (n = 1385)	aRR of delayed surgery
	n (%)	n (%)	n (%)	aRR (95% CI)∗
Age, y				
18-<55	167 (6.5)	94 (8.0)	73 (5.3)	1.00 (Reference)
55-<65	546 (21.3)	270 (22.8)	276 (19.9)	1.15 (0.95-1.39)
65-<75	1107 (43.1)	514 (43.5)	593 (42.8)	1.22 (0.97-1.54)
75-<85	747 (29.1)	304 (25.7)	443 (32.0)	1.29 (1.02-1.64)
Sex				
Female	1539 (60.0)	728 (61.6)	811 (58.6)	1.00 (Reference)
Male	1028 (40.0)	454 (38.4)	574 (41.4)	1.05 (0.98-1.13)
Race/ethnicity				
White	1660 (64.7)	782 (66.2)	878 (63.4)	1.00 (Reference)
Asian	395 (15.4)	178 (15.1)	217 (15.7)	1.14 (1.02-1.27)
Black	183 (7.1)	71 (6.0)	112 (8.1)	1.14 (1.00-1.29)
Hispanic	162 (6.3)	66 (5.6)	96 (6.9)	1.10 (0.97-1.26)
Other	167 (6.5)	85 (7.2)	82 (5.9)	0.96 (0.81-1.12)
NDI, quartile				
1 (least deprived)	645 (25.1)	312 (26.4)	333 (24.0)	1.00 (Reference)
2	633 (24.7)	301 (25.5)	332 (24.0)	0.98 (0.88-1.08)
3	644 (25.1)	304 (25.7)	340 (24.5)	0.98 (0.88-1.09)
4 (most deprived)	645 (25.1)	265 (22.4)	380 (27.4)	1.09 (0.98-1.20)
Insurance type				
Commercial	645 (25.1)	333 (28.2)	312 (22.5)	1.00 (Reference)
Non-Commercial	1922 (74.9)	849 (71.8)	1073 (77.5)	0.98 (0.83-1.17)
Distance to surgery facility, miles				
0-5	473 (18.4)	239 (20.2)	234 (16.9)	1.00 (Reference)
6-10	461 (18.0)	197 (16.7)	264 (19.1)	1.14 (1.01-1.29)
11-25	863 (33.6)	421 (35.6)	442 (31.9)	1.03 (0.92-1.15)
26-50	525 (20.5)	238 (20.1)	287 (20.7)	1.07 (0.95-1.21)
51 or more	245 (9.5)	87 (7.4)	158 (11.4)	1.23 (1.08-1.40)
Charlson Comorbidity Index score				
0	415 (16.2)	214 (18.1)	201 (14.5)	1.00 (Reference)
1-2	1316 (51.3)	631 (53.4)	685 (49.5)	0.96 (0.86-1.08)
3-4	567 (22.1)	233 (19.7)	334 (24.1)	0.99 (0.87-1.13)
5+	269 (10.5)	104 (8.8)	165 (11.9)	0.98 (0.84-1.14)
Smoking history				
No	578 (22.5)	286 (24.2)	292 (21.1)	1.00 (Reference)
Yes	1989 (77.5)	896 (75.8)	1093 (78.9)	1.08 (0.98-1.20)
Additional cancer				
No	2415 (94.1)	1124 (95.1)	1291 (93.2)	1.00 (Reference)
Yes	152 (5.9)	58 (4.9)	94 (6.8)	0.99 (0.87-1.13)
Clinical stage				
I	2258 (88.0)	1022 (86.5)	1236 (89.2)	1.00 (Reference)
II	288 (11.2)	149 (12.6)	139 (10.0)	0.86 (0.76-0.97)
Indeterminate	21 (0.8)	11 (0.9)	10 (0.7)	0.85 (0.56-1.29)
Surgery type				
Bilobectomy/pneumonectomy	43 (1.7)	23 (1.9)	20 (1.4)	1.00 (Reference)
Lobectomy	2132 (83.1)	995 (84.2)	1137 (82.1)	1.07 (0.78-1.47)
Segmentectomy	63 (2.5)	24 (2.0)	39 (2.8)	1.25 (0.86-1.81)
Wedge	329 (12.8)	140 (11.8)	189 (13.6)	1.12 (0.81-1.55)
Surgery year				
2009-2014	1323 (51.5)	580 (49.1)	743 (53.6)	1.00 (Reference)
2015-2019	1244 (48.5)	602 (50.9)	642 (46.4)	1.07 (0.99-1.15)
Number of clinical visits, quartiles				
0-14	595 (23.2)	368 (31.1)	227 (16.4)	1.00 (Reference)
15-18	625 (24.3)	315 (26.6)	310 (22.4)	1.28 (1.13-1.46)
19-24	628 (24.5)	263 (22.3)	365 (26.4)	1.52 (1.35-1.72)
25-78	719 (28.0)	236 (20.0)	483 (34.9)	1.72 (1.53-1.93)

*aRR*, Adjusted relative risk; *NDI*, Neighborhood Deprivation Index.

∗ Results from modified Poisson regression model including age (categorical), sex, race/ethnicity, NDI (quartiles), insurance type, distance to surgery facility, Charlson Comorbidity Index score, smoking history, additional cancer, clinical stage, surgery type, surgery year, and number of clinical visits in past 6 months (quartiles).

### Patient-Level Factors

Patients with delayed (versus timely) surgery were more likely to be older, male, of non-White race/ethnicity, covered by noncommercial insurance, live in more deprived neighborhoods and greater than 50 miles from their surgery facility, have more comorbidities and greater health care use, present with stage I disease, and undergo surgery in 2015-2019 (Table 1). In our multivariable model, several factors were independently associated with increased risk of delayed (vs timely) surgery, specifically age ≥75 years, Black or Asian race, living more than 50 miles from the surgical facility, and greater health care use. The only factor independently associated with reduced risk of delayed surgery was greater-stage disease. In exploratory analyses among patients who underwent surgery from 2015 to 2019, associations were still observed for Black or Asian race, living more than 50 miles from the surgery facility, and greater health care use, but attenuated for age ≥75 years and greater-stage disease (Table E1). Among patients who underwent surgery from 2018 to 2019, only the association with greater health care use reached statistical significance; however, this subcohort was much smaller in size, thereby reducing power to detect associations (Table E2).

**Figure 1:**
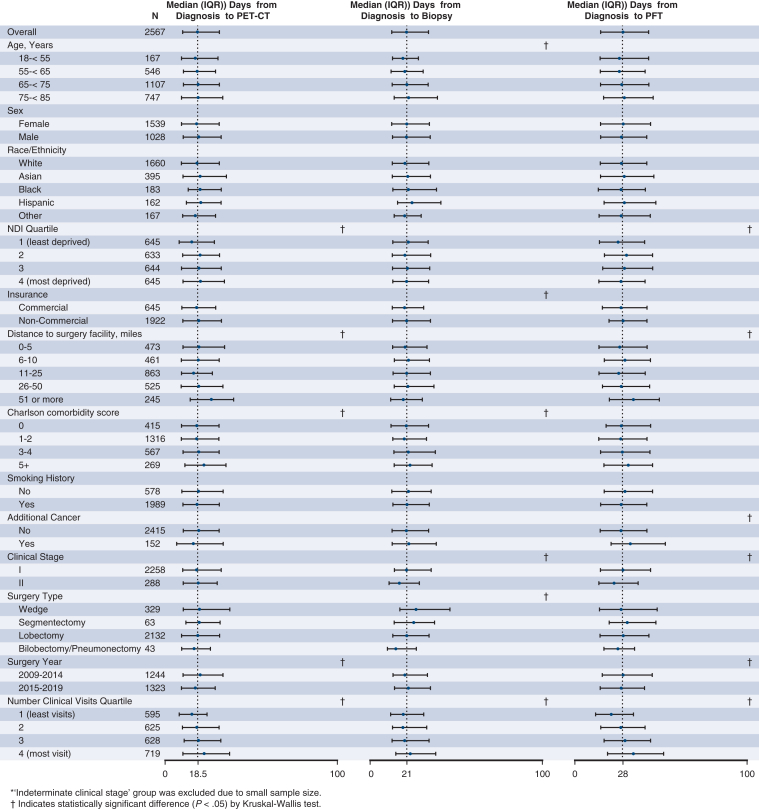

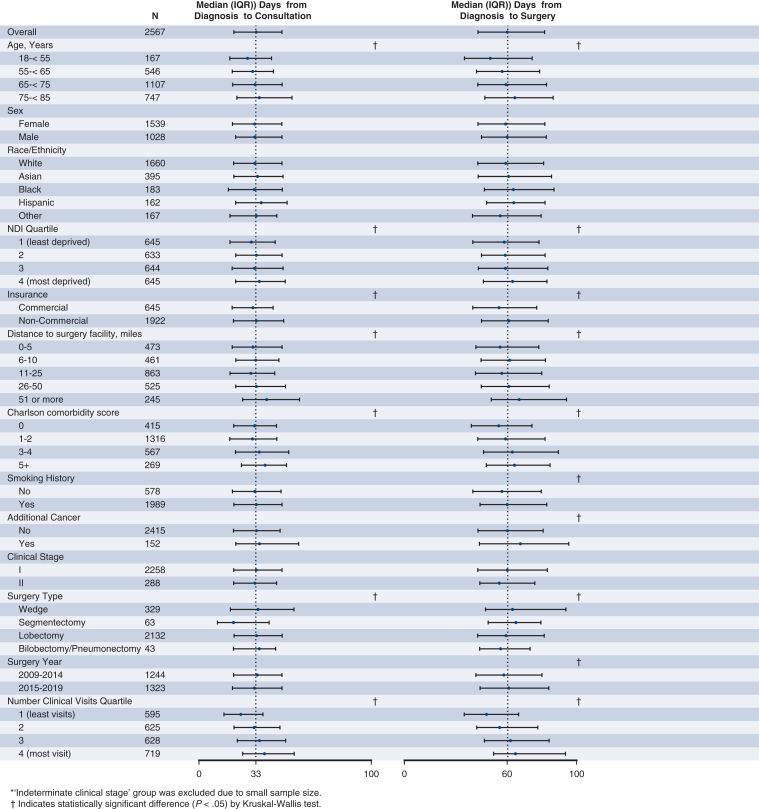
Median time from diagnosis to individual step completion by patient-level factors. *IQR*, Interquartile range; *PET*, positron emission tomography; *CT*, computed tomography; *PFT*, pulmonary function text; *NDI*, Neighborhood Deprivation Index.

As shown in Figure 1, the median time from NSCLC diagnosis to PET-CT, biopsy, PFT, thoracic surgery consultation, and thoracic surgery was 19, 21, 28, 33, and 60 days, respectively, and these time intervals appeared to vary by certain patient-level factors. Specifically, patients with more advanced age, noncommercial insurance, and increasing CCI score all had longer median times for biopsy, thoracic surgery consultation, and thoracic surgery; all of these steps involve an invasive procedure or are intimately associated with procedural planning. Conversely, increased neighborhood deprivation level and driving distance to the surgery facility were each associated with longer median times for noninvasive steps, specifically PET-CT, PFT, and thoracic surgery consultation. Greater health care use was consistently associated with longer median times for all steps. For surgical year, PET-CTs and PFTs were performed more quickly on average in later years (2015-2019), but median time to resection was ultimately longer. Among patients who underwent surgery in the later years, similar patterns in median time to individual step completion by patient-level factors were noted (data not shown).

### Process-Level Factors

Except for thoracic surgery consultation (100% completion for all patients), fewer patients with timely surgery completed each preoperative step. Whereas 68% of patients with delayed surgery completed all 4 preoperative steps (PET-CT, biopsy, PFT, consult), only 54% of those with timely surgery completed all 4 (Table 2). The more steps completed, the longer the median time to surgery, ranging from 41 days for 1 step only to 63 days for all steps. However, step volume alone did not explain timely versus delayed resection. Among those who completed the same step volume and the same combination of steps, the median time to surgery was consistently longer in the delayed surgery group, indicating that each step took longer in the delayed cohort. For those with delayed surgery, the median time to surgery did not differ greatly based on the number of steps performed (beyond 1 preoperative step), whether 2, 3, or 4 steps, indicating a delay at any preoperative step could contribute to delayed surgery. Patients who only underwent PFT and thoracic surgery consult preoperatively had the most protracted median time to surgery at 95 days [IQR, 64-119 days]. Similar findings were again observed among patients who underwent surgery in later years (Table E3 and Table E4).

**Table 2:** Median time to surgery by preoperative step volume and unique step combinations, overall and stratified by timely versus delayed surgery

			Stratified by time to surgery, days				
Preoperative step volume (no steps)	Overall		Timely	Delayed	Timely	Delayed	Δ∗
Unique step combinations (consult | PET/CT | PFT | biopsy)	n (%)	Median [IQR]	n (%)	n (%)	Median [IQR]	Median [IQR]	Median
1 Step†	34 (1%)	41 (24-63)	24 (2%)	10 (1%)	34 (20-42)	72 (65-97)	38
Consult	34 (1%)	41 (24-63)	24 (2%)	10 (1%)	34 (20-42)	72 (65-97)	38
2 Steps†	214 (8%)	48 (34-69)	132 (11%)	82 (6%)	39 (28-47)	81 (64-100)	42
PET/CT + consult	133 (5%)	47 (34-65)	86 (7%)	47 (3%)	38 (26-46)	73 (63-98)	36
Biopsy + consult	31 (1%)	57 (45-82)	15 (1%)	16 (1%)	45 (30-48)	82 (72-99)	37
PFT + consult	50 (2%)	51 (33-81)	31 (3%)	19 (1%)	36 (29-48)	95 (64-119)	59
3 Steps†	731 (28%)	55 (38-78)	385 (33%)	346 (25%)	39 (30-47)	81 (67-106)	42
PET/CT + biopsy + consult	174 (7%)	67 (49-94)	65 (5%)	109 (8%)	43 (34-52)	82 (71-108)	39
PET/CT + PFT + consult	507 (20%)	49 (35-75)	297 (25%)	210 (15%)	37 (29-44)	78 (67-104)	41
PFT + biopsy + consult	50 (2%)	61 (47-78)	23 (2%)	27 (2%)	45 (38-51)	77 (64-109)	32
4 Steps†	1588 (62%)	63 (48-85)	641 (54%)	947 (68%)	45 (37-50)	79 (67-102)	34
PET/CT + PFT + biopsy + consult	1588 (62%)	63 (48-85)	641 (54%)	947 (68%)	45 (37-50)	79 (67-102)	34

*PET*, Positron emission tomography; *CT*, computed tomography; *PFT*, pulmonary function text; *IQR*, interquartile range.

∗ Median difference in days between timely and delayed surgery groups.

† Regardless of which specific steps were completed. Each patient is only represented once across the rows with the footnote “†.”

**Figure 2:**
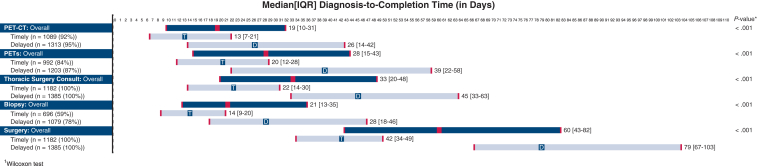
Median time from diagnosis to individual step completion, overall and stratified by timely versus delayed surgery. *IQR*, Interquartile range; *PET*, positron emission tomography; *CT*, computed tomography; *PFT*, pulmonary function text.

However, no individual preoperative step was the bottleneck (Figure 2). Among those who completed a given step, the median time to completion was longer for each preoperative step in the delayed versus timely surgery cohort. Whereas 75% of patients who received timely NSCLC resection completed PET-CT, biopsy, PFT, thoracic surgery consult, and surgical resection in 21, 20, 28, 30 and 49 days, respectively, these intervals were significantly extended in 75% of patients who underwent delayed surgery (42, 46, 58, 63, and 103 days, respectively).

## Discussion

**Figure 3:**
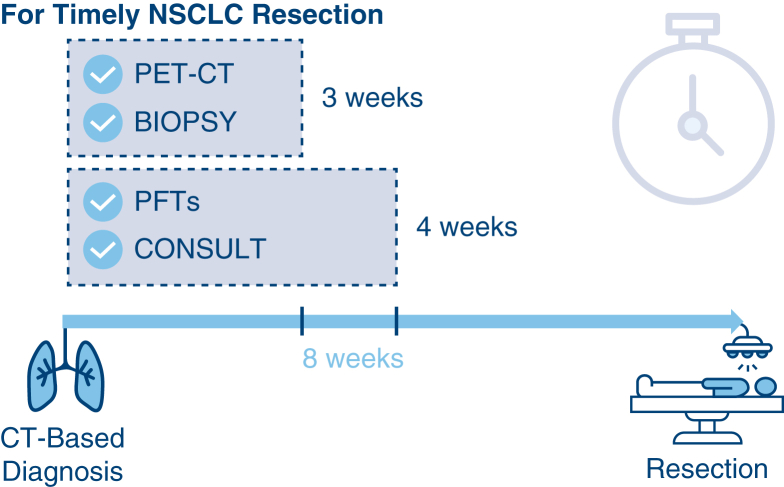
Preliminary benchmarks. *PET*, Positron emission tomography; *CT*, computed tomography; *PFT*, pulmonary function test.

Time-to-treatment initiation for the 8 most common cancers has been steadily increasing in the United States.^2^^,^^3^ For early-stage NSCLC, growing evidence suggests that surgical treatment delayed beyond 8 to 12 weeks of radiographic clinical diagnosis leads to increased cancer recurrence and mortality.^5^^,^^6^ Drivers of delayed treatment and interventions, including quality benchmarks, are less well-defined. Using a closed, integrated care system to evaluate patient- and process-level drivers of delayed care, we found that several factors are associated with delayed curative-intent surgery for early-stage NSCLC, including race/ethnicity and distance to treating facility. In addition, we noted that time to surgery, on average, was longer when more preoperative workup steps occurred. Our time intervals for PET-CT, biopsy, PFTs, and thoracic surgery consultation among patients who received timely surgery can serve as preliminary benchmarks for consensus guidelines on timeliness quality metrics for NSCLC care (Figure 3).

At the patient level, our results are consistent with several NCDB study results.^2^^,^^3^^,^^7^ The time-to-treatment is longer in patients who are older with more comorbidities and stage I disease. The relatively longer time to surgery observed for older patients and those with more comorbidities likely reflects the need for extensive preoperative evaluation to ensure suitability for surgery. Similarly, the association of stage I disease with longer wait times likely reflects prioritization of surgery scheduling for patients with greater-stage disease. Even for stage I disease, recurrence and mortality are greater when surgery is delayed beyond 8 to 12 weeks^5^^,^^6^ (Table E5 and Table E6).

Despite care delivery in an integrated payor-provider system, many concerning nonclinical factors still drive delays. There was a clear trend toward surgical treatment delay for patients experiencing greater socioeconomic deprivation, measured through NDI. Like many studies, we found Black patients were 14% more likely to have delayed surgery compared with White patients. Unlike NCDB studies, we also found that Asian patients were 14% more likely to have delayed surgery. Given NDI is an area-level indicator, we suspect that race/ethnicity may reflect unmeasured individual-level socioeconomic deprivation, as well as structural racism. After accounting for NDI, living more than 50 miles away was also still associated with a 23% increased risk of delayed surgery. In addition to monitoring these care inequities through electronic dashboards, we recommend health care organizations proactively identify patients who are more socioeconomically vulnerable and provide these patients with additional support, including care navigation and mitigation of competing priorities. Although National Cancer Institute–designated and academic centers typically have superior outcomes, it is important to consider that community hospitals have the shortest diagnosis-to-treatment initiation intervals, possibly due to greater continuity of care, geographic proximity, existing insurance contracts and provider availability.^2^^,^^3^^,^^7^, ^8^, ^9^ Community hospitals provide invaluable access and should not be excluded from cancer care pathways. Socioeconomic status, race, and increased distance are consistent, inappropriate drivers of delayed care that require intervention (Figure E1).

Our study also highlights several process-level considerations. More preoperative workup steps increase the time from diagnosis to surgery, with no single step driving delays. Similar to national trends, we find that patients who received care in later years were more likely to experience delayed surgery. The cause is likely multifactorial. New diagnostic and therapeutic discoveries have led to increasingly complex, multistep care, which may ultimately reduce care delivery “effectiveness and efficiency.”^10^ Care consolidation to “centers of excellence,” or in our case, thoracic surgery consolidation to 4 hubs, also likely contributes. In the pursuit of oncologic advances, it is important to remember that effectiveness is only 1 of 6 health care quality domains. Timely surgery can be pursued, in part, by managing the number of preoperative steps through step consolidation (eg, simultaneous tissue and lymph node biopsy through endobronchial ultrasound, or intraoperative tissue biopsy, followed by resection, as appropriate), step reduction (eg, only performing PET-CT when the tumor has an adequate solid component), step automation (eg, reflex biomarker testing after biopsy in the neoadjuvant therapy era), and streamlined communication between specialists through multidisciplinary diagnostic and treatment teams.^4^^,^^11^^,^^12^

Achieving timely surgery within 8 weeks of radiographic diagnosis requires prompt preoperative workup, but additional research characterizing timely step completion is needed. Unfortunately, the NCDB lacks sufficient granularity, and institutional studies are often limited by facility transfer between diagnosis and treatment. Although one claims-based study recommended PET-CT to be performed within 7 days of diagnosis with PET-CT follow-up subsequently within 3 days, followed by treatment within 10 days of follow-up, these condensed intervals are unrealistic.^13^ Conversely, we can clearly delineate appropriate time intervals in a large closed, integrated health care system with negligible loss-to-follow-up. For example, our results highlight that those who underwent timely surgery had a median PET-CT completion time of 13 days (approximately 2 weeks) after CT-based diagnosis and 75% were completed within 3 weeks. Those with timely surgery had their thoracic surgery consult within a median of 3 weeks and 75% had it within 30 days of radiographic diagnosis.

Our results can be foundational for establishing timeliness quality benchmarks for payor and provider organizations. Scoping reviews identify these standardized metrics as essential to reducing delays in care, but current guidelines are sparse.^14^ In the last 2 decades, Dutch consensus guidelines recommended diagnosis and staging workup be completed within 3 weeks and definitive treatment started within 5 weeks after the first pulmonology visit (2006),^15^ whereas guidelines from the United Kingdom recommended that patients with suspected lung cancer receive a specialist appointment in 2 weeks and initiate treatment within 4 weeks of referral (2011 and 2017).^14^ Intricate diagnostic, staging and treatment pathways with associated time points are provided by the UK's National Optimal Lung Cancer Pathway guide, but complexity limits utility for quality improvement.^16^ Our results support simplified, evidence-based benchmarking. Timeliness benchmarks will become increasingly important with increased lung cancer screening uptake in the context of an increasingly complex health system. In particular, establishing these benchmarks for payors could help counter prior authorization delays and protect against uncoordinated managed care networks, where facility transfer between diagnosis and treatment increases the median time to treatment by 11.1 days.^3^ Patients need clear care pathways from primary to secondary to tertiary care.

At present, any timeliness benchmarking is largely aspirational, given interorganizational data collection challenges. However, we recommend professional societies and consortiums, such as the Commission on Cancer, consider adopting timeliness guidelines to reinforce the importance of timely care. To reinforce equitable care, these temporal targets should be consistent irrespective of practice setting or insurance plan. On the basis of our data, we propose that health systems aim to complete PET-CT and biopsy within 3 weeks and PFTs and thoracic surgery consult within 4 weeks after CT-based diagnosis for patients with early-stage NSCLC.

### Limitations

Our characterization of potential drivers of delayed care is not exhaustive. We present the most salient preoperative clinical steps to facilitate benchmarking, but other variably-occurring steps, such as medical clearance, are not included. In particular, individual provider-level factors and inter-organizational factors, such as coordination between discrete provider, payor, and other administrative organizations, are not examined. Our study setting does not need to contend with interorganizational transfers, including fragmented health information exchange between organizations, and we acknowledge that payment structures, which can either disincentivize or reinforce coordination between organizations and multidisciplinary providers, vary between institutions. As an integrated payor-provider system, our study setting is not generalizable to the U.S. health care system; however, this is precisely why it is an ideal setting to generate timeliness benchmarks with complete longitudinal data from diagnosis to treatment. For care delivery that is fragmented across organizations, timeliness benchmarks could reinforce the importance of improving interorganizational care coordination, including through seamless health information exchange and financial incentivizes to deliver coordinated multidisciplinary care across providers and organizations. Diagnosis to treatment initiation should be accomplished within 8-12 weeks for early-stage operative candidates irrespective of organizational complexity or administrative burden to achieve optimal outcomes. This study offers a roadmap with interval metrics on how to accomplish timely treatment of operative early-stage NSCLC.

## Conclusions

Robust evidence indicates that surgical treatment delayed beyond 8 to 12 weeks is associated with increased cancer recurrence and mortality for patients with early-stage NSCLC. Several patient-level factors, such as race/ethnicity, socioeconomic deprivation, and driving distance from residence to hospital, contribute to delays, and these indefensible disparities should be actively recognized and mitigated. As treatment options and health system complexity increase, we need to track timeliness of treatment as a key health care quality indicator. Our results offer preliminary benchmarks to hold orchestrators of health care, including payors, accountable to timely NSCLC management.

## Conflict of Interest Statement

L.C.S. has research funding from AstraZeneca, the National Cancer Institute, and California Tobacco-Related Disease Research Program, unrelated to this work, which has been awarded directly to her institution. J.B.V. has research funding from AstraZeneca, unrelated to this work, which has been awarded directly to his institution. A.L.C has research funding from Amgen, AstraZeneca, Novartis, Roche/Genetech, and Tempus, unrelated to this work, which has been awarded directly to her institution. All other authors reported no conflicts of interest.

The *Journal* policy requires editors and reviewers to disclose conflicts of interest and to decline handling or reviewing manuscripts for which they may have a conflict of interest. The editors and reviewers of this article have no conflicts of interest.

## Figures and Tables

**Table E1:** Risk of delayed surgery for early-stage NSCLC associated with patient-level factors: 2015-2019

Variable	Overall (N = 1323)	<8 weeks (timely) (n = 580)	>8 weeks (delayed) (n = 743)	aRR of delayed surgery
	n (%)	n (%)	n (%)	aRR (95% CI)∗
Age, y				
18-<55	80 (6.0)	43 (7.4)	37 (5.0)	1.00 (Reference)
55-<65	273 (20.6)	136 (23.4)	137 (18.4)	1.08 (0.82-1.41)
65-<75	592 (44.7)	258 (44.5)	334 (45.0)	1.22 (0.88-1.68)
75-<85	378 (28.6)	143 (24.7)	235 (31.6)	1.28 (0.92-1.77)
Sex				
Female	824 (62.3)	367 (63.3)	457 (61.5)	1.00 (Reference)
Male	499 (37.7)	213 (36.7)	286 (38.5)	1.00 (0.91-1.11)
Race/ethnicity				
White	834 (63.0)	386 (66.6)	448 (60.3)	1.00 (Reference)
Asian	202 (15.3)	80 (13.8)	122 (16.4)	1.23 (1.07-1.42)
Black	112 (8.5)	41 (7.1)	71 (9.6)	1.19 (1.01-1.39)
Hispanic	95 (7.2)	39 (6.7)	56 (7.5)	1.12 (0.94-1.33)
Other	80 (6.0)	34 (5.9)	46 (6.2)	1.11 (0.91-1.37)
NDI, quartile				
1 (least deprived)	355 (26.8)	157 (27.1)	198 (26.6)	1.00 (Reference)
2	318 (24.0)	139 (24.0)	179 (24.1)	0.96 (0.84-1.10)
3	310 (23.4)	147 (25.3)	163 (21.9)	0.90 (0.78-1.04)
4 (most deprived)	340 (25.7)	137 (23.6)	203 (27.3)	1.03 (0.90-1.17)
Insurance type				
Commercial	310 (23.4)	158 (27.2)	152 (20.5)	1.00 (Reference)
Noncommercial	1013 (76.6)	422 (72.8)	591 (79.5)	0.97 (0.77-1.22)
Distance to surgery facility, miles				
0-5	154 (11.6)	72 (12.4)	82 (11.0)	1.00 (Reference)
6-10	160 (12.1)	65 (11.2)	95 (12.8)	1.13 (0.93-1.37)
11-25	512 (38.7)	242 (41.7)	270 (36.3)	1.03 (0.87-1.22)
26-50	341 (25.8)	148 (25.5)	193 (26.0)	1.09 (0.92-1.29)
51 or more	156 (11.8)	53 (9.1)	103 (13.9)	1.27 (1.05-1.53)
Charlson Comorbidity Index score				
0	93 (7.0)	50 (8.6)	43 (5.8)	1.00 (Reference)
1-2	712 (53.8)	333 (57.4)	379 (51.0)	1.05 (0.82-1.34)
3-4	362 (27.4)	142 (24.5)	220 (29.6)	1.10 (0.85-1.42)
5+	156 (11.8)	55 (9.5)	101 (13.6)	1.11 (0.85-1.45)
Smoking history				
No	319 (24.1)	147 (25.3)	172 (23.1)	1.00 (Reference)
Yes	1004 (75.9)	433 (74.7)	571 (76.9)	1.09 (0.96-1.24)
Additional cancer				
No	1235 (93.3)	546 (94.1)	689 (92.7)	1.00 (Reference)
Yes	88 (6.7)	34 (5.9)	54 (7.3)	0.98 (0.82-1.16)
Clinical stage				
I	1166 (88.1)	512 (88.3)	654 (88.0)	1.00 (Reference)
II	145 (11.0)	64 (11.0)	81 (10.9)	0.97 (0.83-1.12)
Indeterminate	12 (0.9)	4 (0.7)	8 (1.1)	1.09 (0.74-1.62)
Surgery type				
Bilobectomy/pneumonectomy	19 (1.4)	8 (1.4)	11 (1.5)	1.00 (Reference)
Lobectomy	1052 (79.5)	468 (80.7)	584 (78.6)	0.92 (0.62-1.36)
Segmentectomy	60 (4.5)	23 (4.0)	37 (5.0)	1.04 (0.67-1.62)
Wedge	192 (14.5)	81 (14.0)	111 (14.9)	0.94 (0.62-1.41)
Number of clinical visits, quartiles				
0-14	303 (22.9)	178 (30.7)	125 (16.8)	1.00 (Reference)
15-18	324 (24.5)	148 (25.5)	176 (23.7)	1.31 (1.11-1.54)
19-24	315 (23.8)	129 (22.2)	186 (25.0)	1.40 (1.19-1.65)
25-78	381 (28.8)	125 (21.6)	256 (34.5)	1.57 (1.34-1.84)

*aRR*, Adjusted relative risk; *NDI*, Neighborhood Deprivation Index.

∗ Results from modified Poisson regression model including age (categorical), sex, race/ethnicity, NDI (quartiles), insurance type, distance to surgery facility, Charlson Comorbidity Index score, smoking history, additional cancer, clinical stage, surgery type, and number of clinical visits in past 6 months (quartiles).

**Table E2:** Risk of delayed surgery for early-stage NSCLC associated with patient-level factors: 2018-2019

Variable	Overall (N = 532)	<8 weeks (timely) (n = 162)	>8 weeks (delayed) (n = 370)	aRR of delayed surgery
	n (%)	n (%)	n (%)	aRR (95% CI)∗
Age, y				
18-<55	29 (5.5)	16 (9.9)	13 (3.5)	1.00 (Reference)
55-<65	112 (21.1)	40 (24.7)	72 (19.5)	1.29 (0.81-2.04)
65-<75	232 (43.6)	69 (42.6)	163 (44.1)	1.53 (0.93-2.53)
75-<85	159 (29.9)	37 (22.8)	122 (33.0)	1.62 (0.97-2.68)
Sex				
Female	338 (63.5)	108 (66.7)	230 (62.2)	1.00 (Reference)
Male	194 (36.5)	54 (33.3)	140 (37.8)	1.01 (0.90-1.14)
Race/ethnicity				
White	326 (61.3)	97 (59.9)	229 (61.9)	1.00 (Reference)
Asian	81 (15.2)	19 (11.7)	62 (16.8)	1.15 (0.99-1.33)
Black	55 (10.3)	18 (11.1)	37 (10.0)	0.99 (0.81-1.21)
Hispanic	42 (7.9)	17 (10.5)	25 (6.8)	0.88 (0.69-1.14)
Other	28 (5.3)	11 (6.8)	17 (4.6)	0.87 (0.64-1.17)
NDI, quartile				
1 (least deprived)	164 (30.8)	48 (29.6)	116 (31.4)	1.00 (Reference)
2	109 (20.5)	29 (17.9)	80 (21.6)	0.98 (0.84-1.14)
3	125 (23.5)	39 (24.1)	86 (23.2)	0.97 (0.83-1.14)
4 (most deprived)	134 (25.2)	46 (28.4)	88 (23.8)	0.93 (0.79-1.10)
Insurance type				
Commercial	114 (21.4)	43 (26.5)	71 (19.2)	1.00 (Reference)
Noncommercial	418 (78.6)	119 (73.5)	299 (80.8)	0.84 (0.65-1.10)
Distance to surgery facility, miles				
0-5	72 (13.5)	24 (14.8)	48 (13.0)	1.00 (Reference)
6-10	59 (11.1)	16 (9.9)	43 (11.6)	1.08 (0.86-1.35)
11-25	193 (36.3)	62 (38.3)	131 (35.4)	1.00 (0.82-1.21)
26-50	135 (25.4)	40 (24.7)	95 (25.7)	1.02 (0.84-1.25)
51 or more	73 (13.7)	20 (12.3)	53 (14.3)	1.08 (0.87-1.34)
Charlson Comorbidity Index score				
0	26 (4.9)	15 (9.3)	11 (3.0)	1.00 (Reference)
1-2	274 (51.5)	86 (53.1)	188 (50.8)	1.35 (0.83-2.20)
3-4	171 (32.1)	48 (29.6)	123 (33.2)	1.35 (0.82-2.23)
5+	61 (11.5)	13 (8.0)	48 (13.0)	1.45 (0.87-2.39)
Smoking history				
No	142 (26.7)	49 (30.2)	93 (25.1)	1.00 (Reference)
Yes	390 (73.3)	113 (69.8)	277 (74.9)	1.06 (0.91-1.23)
Additional cancer				
No	494 (92.9)	149 (92.0)	345 (93.2)	1.00 (Reference)
Yes	38 (7.1)	13 (8.0)	25 (6.8)	0.86 (0.69-1.07)
Clinical stage				
I	468 (88.0)	141 (87.0)	327 (88.4)	1.00 (Reference)
II	58 (10.9)	19 (11.7)	39 (10.5)	0.92 (0.77-1.11)
Indeterminate	6 (1.1)	2 (1.2)	4 (1.1)	1.08 (0.65-1.79)
Surgery type				
Bilobectomy/pneumonectomy	3 (0.6)	1 (0.6)	2 (0.5)	1.00 (Reference)
Lobectomy	428 (80.5)	132 (81.5)	296 (80.0)	0.80 (0.41-1.56)
Segmentectomy	27 (5.1)	8 (4.9)	19 (5.1)	0.81 (0.39-1.65)
Wedge	74 (13.9)	21 (13.0)	53 (14.3)	0.81 (0.41-1.59)
Number of clinical visits, quartiles				
0-14	97 (18.2)	44 (27.2)	53 (14.3)	1.00 (Reference)
15-18	123 (23.1)	41 (25.3)	82 (22.2)	1.21 (0.97-1.50)
19-24	130 (24.4)	36 (22.2)	94 (25.4)	1.33 (1.07-1.65)
25-78	182 (34.2)	41 (25.3)	141 (38.1)	1.39 (1.14-1.70)

*aRR,* Adjusted relative risk; *NDI*, Neighborhood Deprivation Index.

∗ Results from modified Poisson regression model including age (categorical), sex, race/ethnicity, NDI (quartiles), insurance type, distance to surgery facility, Charlson Comorbidity Index score, smoking history, additional cancer, clinical stage, surgery type, and number of clinical visits in past 6 months (quartiles).

**Table E3:** Median time to surgery by preoperative step volume and unique step combinations, overall and stratified by timely versus delayed surgery: 2015-2019 and 2018-2019

			Stratified by time to surgery, days				
Preoperative step volume (no steps)	Overall		Timely	Delayed	Timely	Delayed	Δ∗
Unique step combinations (consult | PET/CT | PFT | biopsy)	n (%)	Median [IQR]	n (%)	n (%)	Median [IQR]	Median [IQR]	Median
Time period: 2015-2019							
1 Step†	20 (2%)	46 (31-66)	12 (2%)	8 (1%)	34 (22-40)	79 (64-99)	45
Consult	20 (2%)	46 (31-66)	12 (2%)	8 (1%)	34 (22-40)	79 (64-99)	45
2 Steps†	101 (8%)	47 (35-73)	66 (11%)	35 (5%)	39 (31-47)	83 (71-101)	44
PET/CT + consult	57 (4%)	43 (34-63)	41 (7%)	16 (2%)	39 (33-46)	78 (68-92)	39
Biopsy + consult	17 (1%)	57 (45-81)	8 (1%)	9 (1%)	43 (34-48)	81 (75-87)	38
PFT + consult	27 (2%)	51 (31-98)	17 (3%)	10 (1%)	35 (26-48)	112 (96-126)	77
3 Steps†∗	403 (30%)	55 (39-78)	207 (36%)	196 (26%)	40 (30-48)	78 (67-104)	38
PET/CT + biopsy + consult	72 (5%)	68 (51-85)	27 (5%)	45 (6%)	46 (36-53)	81 (71-107)	35
PET/CT + PFT + consult	309 (23%)	51 (37-76)	173 (30%)	136 (18%)	39 (30-47)	78 (67-104)	39
PFT + biopsy + consult	22 (2%)	63 (47-81)	7 (1%)	15 (2%)	41 (38-47)	77 (63-89)	36
4 Steps†	799 (60%)	66 (49-88)	295 (51%)	504 (68%)	44 (37-51)	79 (68-102)	35
PET/CT + PFT + biopsy + consult	799 (60%)	66 (49-88)	295 (51%)	504 (68%)	44 (37-51)	79 (68-102)	35
Time period: 2018-2019							
1 Step†	6 (1%)	40 (38-62)	4 (2%)	2 (1%)	38 (34-40)	110 (62-158)	72
Consult	6 (1%)	40 (38-62)	4 (2%)	2 (1%)	38 (34-40)	110 (62-158)	72
2 Steps†	47 (9%)	51 (34-80)	28 (17%)	19 (5%)	37 (30-48)	82 (69-98)	45
PET/CT + consult	26 (5%)	47 (34-69)	16 (10%)	10 (3%)	37 (24-43)	81 (68-87)	45
Biopsy + consult	10 (2%)	66 (48-81)	4 (2%)	6 (2%)	47 (39-52)	80 (75-86)	34
PFT + consult	11 (2%)	47 (29-96)	8 (5%)	3 (1%)	34 (27-49)	98 (96-126)	65
3 Steps†	169 (32%)	71 (50-89)	58 (36%)	111 (30%)	43 (35-51)	81 (71-106)	38
PET/CT + biopsy + consult	35 (7%)	71 (54-85)	12 (7%)	23 (6%)	47 (43-55)	77 (71-98)	30
PET/CT + PFT + consult	123 (23%)	71 (49-92)	43 (27%)	80 (22%)	42 (32-49)	84 (71-106)	42
PFT + biopsy + consult	11 (2%)	66 (51-81)	3 (2%)	8 (2%)	45 (38-51)	78 (64-99)	33
4 Steps†	310 (58%)	74 (58-98)	72 (44%)	238 (64%)	48 (42-53)	83 (70-106)	35
PET/CT + PFT + biopsy + consult	310 (58%)	74 (58-98)	72 (44%)	238 (64%)	48 (42-53)	83 (70-106)	35

*PET*, Positron emission tomography; *CT*, computed tomography; *PFT*, pulmonary function text; *IQR*, interquartile range.

∗ Median difference in days between timely and delayed surgery groups.

† Regardless of which specific steps were completed, each patient is only represented once across the rows with the footnote “†.”

**Table E4:** Median time intervals for all preoperative steps completed after diagnosis, overall and stratified by timely versus delayed surgery, during 2009-2019-2015-2019, and 2018-2019

Time interval	Overall			Timely surgery (≤8 weeks)			Delayed surgery (>8 weeks)			*P* value∗
	No completed (%)	Range	Median [IQR]	No completed (%)	Range	Median [IQR]	No completed (%)	Range	Median [IQR]	
Time period: 2009-2019										
Diagnosis to:										
PET-CT	2402 (94%)	0.0-348.0	19 (10-31)	1089 (92%)	0.0-50.0	13 (7-21)	1313 (95%)	0.0-348.0	26 (14-42)	<.001
PFT	2195 (86%)	0.0-349.0	28 (15-43)	992 (84%)	0.0-52.0	20 (12-28)	1203 (87%)	0.0-349.0	39 (22-58)	<.001
Thoracic surgery E-referral request	2148 (84%)	0.0-337.0	24 (14-39)	950 (80%)	0.0-49.0	16 (8-23)	1198 (86%)	0.0-337.0	35 (22-53)	<.001
Thoracic surgery consultation	2567 (100%)	0.0-342.0	33 (20-48)	1182 (100%)	0.0-52.0	22 (14-30)	1385 (100%)	0.0-342.0	45 (33-63)	<.001
Biopsy	1775 (69%)	0.0-331.0	21 (13-35)	696 (59%)	0.0-42.0	14 (9-20)	1079 (78%)	0.0-331.0	28 (18-46)	<.001
EBUS/MED										
Same-day + in-advance EBUS/MED	478 (19%)	1.0-163.0	43 (31-61)	216 (18%)	1.0-56.0	35 (25-43)	262 (19%)	6.0-163.0	59 (41-75)	<.001
In-advance EBUS/MED	285 (11%)	1.0-113.0	39 (27-53)	101 (9%)	1.0-48.0	26 (18-33)	184 (13%)	6.0-113.0	48 (36-63)	<.001
In-advance EBUS	148 (6%)	1.0-95.0	31 (21-47)	53 (4%)	1.0-48.0	21 (14-27)	95 (7%)	6.0-95.0	41 (28-56)	<.001
In-advance MED	137 (5%)	12.0-113.0	43 (33-59)	48 (4%)	12.0-45.0	32 (25-37)	89 (6%)	22.0-113.0	52 (43-67)	<.001
Surgery	2567 (100%)	7.0-358.0	60 (43-82)	1182 (100%)	7.0-56.0	42 (34-49)	1385 (100%)	57.0-358.0	79 (67-103)	<.001
Thoracic surgery E-referral to:										
PFT	1275 (50%)	0.0-615.0	9 (4-16)	596 (50%)	0.0-404.0	7 (3-13)	679 (49%)	0.0-615.0	11 (5-20)	<.001
Consultation	2148 (84%)	0.0-123.0	7 (3-11)	950 (80%)	0.0-41.0	6 (3-8)	1198 (86%)	0.0-123.0	8 (4-13)	<.001
Thoracic surgery consultation to:										
Surgery	2567 (100%)	1.0-211.0	23 (15-36)	1182 (100%)	1.0-53.0	17 (11-25)	1385 (100%)	1.0-211.0	32 (20-49)	<.001
Time period: 2015-2019										
Diagnosis to:										
PET-CT	1237 (93%)	0.0-188.0	17 (9-29)	536 (92%)	0.0-50.0	12 (7-21)	701 (94%)	0.0-188.0	23 (13-39)	<.001
PFT	1157 (87%)	0.0-194.0	27 (15-41)	492 (85%)	0.0-52.0	19 (11-27)	665 (90%)	0.0-194.0	36 (22-54)	<.001
Thoracic surgery E-referral request	1093 (83%)	0.0-237.0	24 (13-39)	452 (78%)	0.0-49.0	16 (7-23)	641 (86%)	0.0-237.0	33 (20-52)	<.001
Thoracic surgery consultation	1323 (100%)	0.0-238.0	32 (19-48)	580 (100%)	0.0-52.0	22 (13-30)	743 (100%)	0.0-238.0	44 (30-62)	<.001
Biopsy	873 (66%)	0.0-175.0	22 (13-37)	312 (54%)	0.0-41.0	14 (10-21)	561 (76%)	2.0-175.0	29 (17-46)	<.001
EBUS/MED										
Same-day + in-advance EBUS/MED	236 (18%)	1.0-134.0	40 (27-59)	87 (15%)	1.0-56.0	27 (17-39)	149 (20%)	6.0-134.0	49 (34-69)	<.001
In-advance EBUS/MED	180 (14%)	1.0-97.0	36 (23-49)	59 (10%)	1.0-48.0	22 (14-28)	121 (16%)	6.0-97.0	43 (31-61)	<.001
In-advance EBUS	126 (10%)	1.0-95.0	30 (21-47)	43 (7%)	1.0-48.0	20 (13-27)	83 (11%)	6.0-95.0	40 (27-57)	<.001
In-advance MED	54 (4%)	12.0-97.0	45 (34-57)	16 (3%)	12.0-41.0	28 (21-36)	38 (5%)	22.0-97.0	49 (43-64)	<.001
Surgery	1323 (100%)	7.0-276.0	61 (44-84)	580 (100%)	7.0-56.0	42 (34-49)	743 (100%)	57.0-276.0	80 (68-102)	<.001
Thoracic surgery E-referral to:										
PFT	640 (48%)	0.0-615.0	8 (4-16)	280 (48%)	0.0-404.0	7 (3-12)	360 (48%)	0.0-615.0	11 (5-20)	<.001
Consultation	1093 (83%)	0.0-123.0	7 (3-12)	452 (78%)	0.0-40.0	6 (3-8)	641 (86%)	0.0-123.0	7 (4-14)	<.001
Thoracic surgery consultation to:										
Surgery	1323 (100%)	1.0-190.0	25 (15-39)	580 (100%)	1.0-53.0	18 (11-25)	743 (100%)	2.0-190.0	34 (21-51)	<.001
Time period: 2018-2019										
Diagnosis to:										
PET-CT	494 (93%)	0.0-188.0	18 (10-31)	143 (88%)	0.0-50.0	11 (0-19)	351 (95%)	0.0-188.0	21 (13-37)	<.001
PFT	455 (86%)	0.0-194.0	29 (16-43)	126 (78%)	0.0-50.0	17 (10-27)	329 (89%)	1.0-194.0	35 (21-49)	<.001
Thoracic surgery E-referral request	447 (84%)	0.0-183.0	25 (11-42)	124 (77%)	0.0-42.0	12 (4-23)	323 (87%)	0.0-183.0	31 (18-50)	<.001
Thoracic surgery consultation	532 (100%)	0.0-196.0	35 (21-53)	162 (100%)	0.0-52.0	17 (9-30)	370 (100%)	1.0-196.0	44 (30-61)	<.001
Biopsy	352 (66%)	0.0-175.0	25 (14-39)	81 (50%)	0.0-39.0	14 (10-25)	271 (73%)	3.0-175.0	29 (17-46)	<.001
EBUS/MED										
Same-day + in-advance EBUS/MED	91 (17%)	1.0-101.0	39 (26-63)	26 (16%)	1.0-55.0	27 (21-37)	65 (18%)	6.0-101.0	47 (29-71)	<.001
In-advance EBUS/MED	77 (14%)	1.0-95.0	34 (24-51)	21 (13%)	1.0-48.0	24 (17-29)	56 (15%)	6.0-95.0	42 (28-63)	<.001
In-advance EBUS	67 (13%)	1.0-95.0	33 (23-51)	19 (12%)	1.0-48.0	23 (16-29)	48 (13%)	6.0-95.0	41 (27-63)	<.001
In-advance MED	10 (2%)	22.0-88.0	42 (34-57)	2 (1%)	28.0-40.0	34 (28-40)	8 (2%)	22.0-88.0	44 (34-64)	<.001
Surgery	532 (100%)	8.0-257.0	71 (53-92)	162 (100%)	8.0-56.0	44 (35-51)	370 (100%)	57.0-257.0	83 (70-105)	<.001
Thoracic surgery E-referral to:										
PFT	250 (47%)	0.0-615.0	11 (5-21)	72 (44%)	0.0-404.0	8 (5-14)	178 (48%)	0.0-615.0	12 (6-22)	<.001
Consultation	447 (84%)	0.0-123.0	7 (3-14)	124 (77%)	0.0-40.0	6 (3-10)	323 (87%)	0.0-123.0	7 (4-15)	<.001
Thoracic surgery consultation to:										
Surgery	532 (100%)	1.0-190.0	33 (19-48)	162 (100%)	1.0-53.0	21 (12-31)	370 (100%)	4.0-190.0	40 (25-55)	<.001

*IQR*, Interquartile range; *PET*, positron emission tomography; *CT*, computed tomography; *PFT*, pulmonary function text; *EBUS/MED*, endobronchial ultrasound/mediastinoscopy.

∗ Wilcoxon test.

**Table E5:** Median time interval for all preoperative steps completed before and after diagnosis, overall and stratified by timely versus delayed surgery, during 2009-2019-2015-2019, and 2018-2019

Time interval	Overall			Timely surgery (≤8 weeks)			Delayed surgery (>8 weeks)			*P* value∗
	No completed (%)	Range	Median [IQR]	No completed (%)	Range	Median [IQR]	No completed (%)	Range	Median [IQR]	
Time period: 2009-2019										
Diagnosis to:										
PET-CT	2408 (94%)	−293.0-348.0	18 (9-31)	1090 (92%)	−293.0-50.0	13 (7-21)	1318 (95%)	−192.0-348.0	26 (13-42)	<.001
PFT	2453 (96%)	−2125.0-349.0	24 (11-41)	1126 (95%)	−2125.0-52.0	18 (8-27)	1327 (96%)	−1435.0-349.0	35 (18-56)	<.001
Thoracic surgery E-referral request	2148 (84%)	0.0-337.0	24 (14-39)	950 (80%)	0.0-49.0	16 (8-23)	1198 (86%)	0.0-337.0	35 (22-53)	<.001
Thoracic surgery consultation	2567 (100%)	0.0-342.0	33 (20-48)	1182 (100%)	0.0-52.0	22 (14-30)	1385 (100%)	0.0-342.0	45 (33-63)	<.001
Biopsy	1775 (69%)	0.0-331.0	21 (13-35)	696 (59%)	0.0-42.0	14 (9-20)	1079 (78%)	0.0-331.0	28 (18-46)	<.001
EBUS/MED										
Same-day + in-advance EBUS/MED	478 (19%)	1.0-163.0	43 (31-61)	216 (18%)	1.0-56.0	35 (25-43)	262 (19%)	6.0-163.0	59 (41-75)	<.001
In-advance EBUS/MED	285 (11%)	1.0-113.0	39 (27-53)	101 (9%)	1.0-48.0	26 (18-33)	184 (13%)	6.0-113.0	48 (36-63)	<.001
In-advance EBUS	148 (6%)	1.0-95.0	31 (21-47)	53 (4%)	1.0-48.0	21 (14-27)	95 (7%)	6.0-95.0	41 (28-56)	<.001
In-advance MED	137 (5%)	12.0-113.0	43 (33-59)	48 (4%)	12.0-45.0	32 (25-37)	89 (6%)	22.0-113.0	52 (43-67)	<.001
Surgery	2567 (100%)	7.0-358.0	60 (43-82)	1182 (100%)	7.0-56.0	42 (34-49)	1385 (100%)	57.0-358.0	79 (67-103)	<.001
Thoracic surgery E-referral to:										
PFT	2046 (80%)	−2132.0-134.0	3 (-9-11)	900 (76%)	−2132.0-46.0	3 (-5-9)	1146 (83%)	−1487.0-134.0	3 (-15-13)	.373
Consultation	2148 (84%)	0.0-123.0	7 (3-11)	950 (80%)	0.0-41.0	6 (3-8)	1198 (86%)	0.0-123.0	8 (4-13)	<.001
Thoracic surgery consultation to:										
Surgery	2567 (100%)	1.0-211.0	23 (15-36)	1182 (100%)	1.0-53.0	17 (11-25)	1385 (100%)	1.0-211.0	32 (20-49)	<.001
Time period: 2015-2019										
Diagnosis to:										
PET-CT	1243 (94%)	−293.0-188.0	17 (9-29)	537 (93%)	−293.0-50.0	12 (7-21)	706 (95%)	−192.0-188.0	22 (13-39)	<.001
PFT	1304 (99%)	−2125.0-194.0	23 (11-39)	568 (98%)	−2125.0-52.0	16 (7-26)	736 (99%)	−1005.0-194.0	33 (16-52)	<.001
Thoracic surgery E-referral request	1093 (83%)	0.0-237.0	24 (13-39)	452 (78%)	0.0-49.0	16 (7-23)	641 (86%)	0.0-237.0	33 (20-52)	<.001
Thoracic surgery consultation	1323 (100%)	0.0-238.0	32 (19-48)	580 (100%)	0.0-52.0	22 (13-30)	743 (100%)	0.0-238.0	44 (30-62)	<.001
Biopsy	873 (66%)	0.0-175.0	22 (13-37)	312 (54%)	0.0-41.0	14 (10-21)	561 (76%)	2.0-175.0	29 (17-46)	<.001
EBUS/MED										
Same-day + in-advance EBUS/MED	236 (18%)	1.0-134.0	40 (27-59)	87 (15%)	1.0-56.0	27 (17-39)	149 (20%)	6.0-134.0	49 (34-69)	<.001
In-advance EBUS/MED	180 (14%)	1.0-97.0	36 (23-49)	59 (10%)	1.0-48.0	22 (14-28)	121 (16%)	6.0-97.0	43 (31-61)	<.001
In-advance EBUS	126 (10%)	1.0-95.0	30 (21-47)	43 (7%)	1.0-48.0	20 (13-27)	83 (11%)	6.0-95.0	40 (27-57)	<.001
In-advance MED	54 (4%)	12.0-97.0	45 (34-57)	16 (3%)	12.0-41.0	28 (21-36)	38 (5%)	22.0-97.0	49 (43-64)	<.001
Surgery	1323 (100%)	7.0-276.0	61 (44-84)	580 (100%)	7.0-56.0	42 (34-49)	743 (100%)	57.0-276.0	80 (68-102)	<.001
Thoracic surgery E-referral to:										
PFT	1075 (81%)	−2132.0-134.0	2 (-11-10)	440 (76%)	−2132.0-46.0	2 (-6-8)	635 (85%)	−1023.0-134.0	2 (-15-12)	<.001
Consultation	1093 (83%)	0.0-123.0	7 (3-12)	452 (78%)	0.0-40.0	6 (3-8)	641 (86%)	0.0-123.0	7 (4-14)	<.001
Thoracic surgery consultation to:										
Surgery	1323 (100%)	1.0-190.0	25 (15-39)	580 (100%)	1.0-53.0	18 (11-25)	743 (100%)	2.0-190.0	34 (21-51)	<.001
Time period: 2018-2019										
Diagnosis to:										
PET-CT	498 (94%)	−293.0-188.0	18 (9-31)	144 (89%)	−293.0-50.0	11 (0-19)	354 (96%)	−82.0-188.0	21 (13-37)	<.001
PFT	524 (98%)	−2125.0-194.0	25 (11-41)	156 (96%)	−2125.0-50.0	13 (4-23)	368 (99%)	−848.0-194.0	32 (16-48)	<.001
Thoracic surgery E-referral request	447 (84%)	0.0-183.0	25 (11-42)	124 (77%)	0.0-42.0	12 (4-23)	323 (87%)	0.0-183.0	31 (18-50)	<.001
Thoracic surgery consultation	532 (100%)	0.0-196.0	35 (21-53)	162 (100%)	0.0-52.0	17 (9-30)	370 (100%)	1.0-196.0	44 (30-61)	<.001
Biopsy	352 (66%)	0.0-175.0	25 (14-39)	81 (50%)	0.0-39.0	14 (10-25)	271 (73%)	3.0-175.0	29 (17-46)	<.001
EBUS/MED										
Same-day + in-advance EBUS/MED	91 (17%)	1.0-101.0	39 (26-63)	26 (16%)	1.0-55.0	27 (21-37)	65 (18%)	6.0-101.0	47 (29-71)	<.001
In-advance EBUS/MED	77 (14%)	1.0-95.0	34 (24-51)	21 (13%)	1.0-48.0	24 (17-29)	56 (15%)	6.0-95.0	42 (28-63)	<.001
In-advance EBUS	67 (13%)	1.0-95.0	33 (23-51)	19 (12%)	1.0-48.0	23 (16-29)	48 (13%)	6.0-95.0	41 (27-63)	<.001
In-advance MED	10 (2%)	22.0-88.0	42 (34-57)	2 (1%)	28.0-40.0	34 (28-40)	8 (2%)	22.0-88.0	44 (34-64)	<.001
Surgery	532 (100%)	8.0-257.0	71 (53-92)	162 (100%)	8.0-56.0	44 (35-51)	370 (100%)	57.0-257.0	83 (70-105)	<.001
Thoracic surgery E-referral to:										
PFT	439 (83%)	−2132.0-134.0	2 (-15-12)	118 (73%)	−2132.0-46.0	3 (-10-10)	321 (87%)	−854.0-134.0	2 (-17-12)	<.001
Consultation	447 (84%)	0.0-123.0	7 (3-14)	124 (77%)	0.0-40.0	6 (3-10)	323 (87%)	0.0-123.0	7 (4-15)	<.001
Thoracic surgery consultation to:										
Surgery	532 (100%)	1.0-190.0	33 (19-48)	162 (100%)	1.0-53.0	21 (12-31)	370 (100%)	4.0-190.0	40 (25-55)	<.001

*IQR*, Interquartile range; *PET*, positron emission tomography; *CT*, computed tomography; *PFT*, pulmonary function text; *EBUS/MED*, endobronchial ultrasound/mediastinoscopy.

∗ Wilcoxon test.

**Table E6:** Proportion of patients receiving preoperative steps completed before and after diagnosis, overall and stratified by timely versus delayed surgery, during 2009-2019-2015-2019, and 2018-2019

Preoperative step	Overall	Timely (≤8 weeks)	Delayed (>8 weeks)	*P* value∗
	n (%)	n (%)	n (%)	
Time period: 2009-2019				
Received PFT	2453 (96%)	1126 (95%)	1327 (96%)	.5
Received referral	2148 (84%)	950 (80%)	1198 (86%)	<.001
Received PET	2408 (94%)	1090 (92%)	1318 (95%)	.002
Received tissue biopsy	1775 (69%)	696 (59%)	1079 (78%)	<.001
Received EBUS/MED	478 (19%)	216 (18%)	262 (19%)	.677
Received in-advance EBUS/MED	285 (11%)	101 (9%)	184 (13%)	<.001
Time period: 2015-2019				
Received PFT	1304 (99%)	568 (98%)	736 (99%)	.087
Received referral	1093 (83%)	452 (78%)	641 (86%)	<.001
Received PET	1243 (94%)	537 (93%)	706 (95%)	.065
Received tissue biopsy	873 (66%)	312 (54%)	561 (76%)	<.001
Received EBUS/MED	236 (18%)	87 (15%)	149 (20%)	.017
Received in-advance EBUS/MED	180 (14%)	59 (10%)	121 (16%)	.001
Time period: 2018-2019				
Received PFT	524 (98%)	156 (96%)	368 (99%)	.006
Received referral	447 (84%)	124 (77%)	323 (87%)	.002
Received PET	498 (94%)	144 (89%)	354 (96%)	.003
Received tissue biopsy	352 (66%)	81 (50%)	271 (73%)	<.001
Received EBUS/MED	91 (17%)	26 (16%)	65 (18%)	.669
Received in-advance EBUS/MED	77 (14%)	21 (13%)	56 (15%)	.512

*PFT*, Pulmonary function text; *PET*, positron emission tomography; *EBUS/MED*, endobronchial ultrasound/mediastinoscopy.

∗ Pearson χ^2^ test.
